# Chitotriosidase, a biomarker of amyotrophic lateral sclerosis, accentuates neurodegeneration in spinal motor neurons through neuroinflammation

**DOI:** 10.1186/s12974-020-01909-y

**Published:** 2020-08-06

**Authors:** Anu Mary Varghese, Mausam Ghosh, Savita Kumari Bhagat, K. Vijayalakshmi, Veeramani Preethish-Kumar, Seena Vengalil, Pradeep-Chandra-Reddy Chevula, Saraswati Nashi, Kiran Polavarapu, Meenakshi Sharma, Rupinder Singh Dhaliwal, Mariamma Philip, Atchayaram Nalini, Phalguni Anand Alladi, Talakad N. Sathyaprabha, Trichur R. Raju

**Affiliations:** 1 Department of Neurophysiology, National Institute of Mental Health and Neuro Sciences, Hosur Road, Bengaluru, 560 029 India; 2 Department of Clinical Neuroscience, National Institute of Mental Health and Neuro Sciences, Hosur Road, Bengaluru, 560 029 India; 3 Department of Neurology, National Institute of Mental Health and Neuro Sciences, Hosur Road, Bengaluru, 560 029 India; 4 Division of Non Communicable Disease, Indian Council of Medical Research, New Delhi, India; 5 Department of Biostatistics, National Institute of Mental Health and Neuro Sciences, Hosur Road, Bengaluru, 560 029 India; 6 Department of Clinical Pharmacology & Neurotoxicology, National Institute of Mental Health and Neuro Sciences, Hosur Road, Bengaluru, 560 029 India

**Keywords:** Chitotriosidase, Amyotrophic lateral sclerosis, Biomarker, Neuroinflammation, Glia, Neurodegeneration

## Abstract

**Background:**

Cerebrospinal fluid from amyotrophic lateral sclerosis patients (ALS-CSF) induces neurodegenerative changes in motor neurons and gliosis in sporadic ALS models. Search for identification of toxic factor(s) in CSF revealed an enhancement in the level and enzyme activity of chitotriosidase (CHIT-1). Here, we have investigated its upregulation in a large cohort of samples and more importantly its role in ALS pathogenesis in a rat model.

**Methods:**

CHIT-1 level in CSF samples from ALS (*n* = 158), non-ALS (*n* = 12) and normal (*n* = 48) subjects were measured using ELISA. Enzyme activity was also assessed (ALS, *n* = 56; non-ALS, *n* = 10 and normal-CSF, *n* = 45). Recombinant CHIT-1 was intrathecally injected into Wistar rat neonates. Lumbar spinal cord sections were stained for Iba1, glial fibrillary acidic protein and choline acetyl transferase to identify microglia, astrocytes and motor neurons respectively after 48 h of injection. Levels of tumour necrosis factor-α and interleukin-6 were measured by ELISA.

**Findings:**

CHIT-1 level in ALS-CSF samples was increased by 20-fold and it can distinguish ALS patients with a sensitivity of 87% and specificity of 83.3% at a cut off level of 1405.43 pg/ml. Enzyme activity of CHIT-1 was also 15-fold higher in ALS-CSF and has a sensitivity of 80.4% and specificity of 80% at cut off value of 0.1077989 μmol/μl/min. Combining CHIT-1 level and activity together gave a positive predictive value of 97.78% and negative predictive value of 100%. Administration of CHIT-1 increased microglial numbers and astrogliosis in the ventral horn with a concomitant increase in the levels of pro-inflammatory cytokines. Amoeboid-shaped microglial and astroglial cells were also present around the central canal. CHIT-1 administration also resulted in the reduction of motor neurons.

**Conclusions:**

CHIT-1, an early diagnostic biomarker of sporadic ALS, activates glia priming them to attain a toxic phenotype resulting in neuroinflammation leading to motor neuronal death.

## Background

Diagnosis of amyotrophic lateral sclerosis (ALS), a non-cell autonomous neurodegenerative disorder marked by degeneration of motor neurons along with muscular atrophy, is still challenging as the symptom development could be delayed during initial phase and may overlap with other neurodegenerative disorders [1–4]. Biomarkers can play a pivotal role in early and accurate diagnosis of neurodegenerative disorders including ALS. We were the first to report that chitotriosidase (CHIT-1) can be a putative biomarker for ALS, as the level and activity of CHIT-1 was markedly increased in cerebrospinal fluid of ALS patients (ALS-CSF) [5].

We have shown that CHIT-1 is exclusively expressed by microglial cells in the CNS, and its expression is augmented when they are exposed to ALS-CSF [5]. CHIT-1 was found to be co-localised with microglia in the spinal cord of ALS patients [6]. CHIT-1 belonging to the class of 18 glycosyl hydrolases cleaves its natural substrate chitin into oligosaccharides and monosaccharide glucosamines [7]. However, the consequence of its activity, its presence in the CSF and its exact role in ALS pathogenesis remains to be determined. In the reported literature, there is inadequate clarity about the role of CHIT-1. While some studies suggest that it is toxic, others assign it a protective role in the human system [8, 9].

CHIT-1 is reported to be upregulated in several neurological conditions like acute ischemic stroke, Alzheimer’s disease, cerebral adrenoleukodystrophy and cerebrovascular dementia [10–12]. It is also increased in multiple sclerosis, a neuroinflammatory condition [13]. However, the level of CHIT-1 in ALS patients is several folds higher than that reported in other neurological disorders [14–18]. We undertook this study to assess the levels and enzyme activity of CHIT-1 in a larger cohort of samples to validate its utility as a possible biomarker for ALS. We have also attempted to determine the role played by CHIT-1 in the pathogenesis of ALS in a rat model developed in our laboratory, which will enable identification of potential therapeutic targets.

## Materials and methods

### CSF collection

CSF samples were collected after obtaining informed consent as per the institutional human ethics committee guidelines. CSF samples of ALS patients diagnosed using El Escorial criteria [19] were obtained through lumbar puncture. ALS Functional Rating Scale (ALS-FRS) was done to determine the severity of the disease at the time of sample collection. CSF from age- and gender-matched patients with non-neurodegenerative, non-infectious neurological diseases such as benign intracranial hypertension and peripheral neuropathy were also collected and used as non-ALS (NALS-CSF) samples, while CSF samples of patients undergoing orthopaedic surgery without any neurological involvement were used as normal CSF (N-CSF). Intrathecal procedure was carried out by neurologists and orthopaedic surgeons under aseptic condition. CSF samples were snap frozen in liquid nitrogen and stored at − 80 °C until further use. Table 1 provides details of CSF samples used for the study.

**Table 1:** Demographic details of CSF samples used in the study

Characteristic	N-CSF	NALS-CSF	ALS-CSF
Age (mean ± SD)	42.88 ± 8.25	52.34 ± 8.78	50.82 ± 9.57
Sex (F/M)	18/30	5/7	36/122
Disease duration (months)	NA	NA	14.55 ± 12.96
Onset (bulbar/limb)	NA	NA	57/101
ALS-FRS score	NA	NA	28.37 ± 7.28

*N-CSF* normal CSF, *NALS-CSF* non-ALS-CSF, *ALS-CSF* CSF of amyotrophic lateral sclerosis patients, *ALS-FRS score* ALS functional rating scale score, *NA* not applicable

### Enzyme-linked immunosorbent assay (ELISA) and enzyme activity of CHIT-1

ELISA for CSF (48 N-CSF, 12 NALS and 158 ALS-CSF) was performed using commercially available kit for CHIT-1 (MBL International, USA) according to manufacturer’s protocol. The CHIT-1 enzyme activity was measured in 45 N-CSF, 10 NALS-CSF and 56 ALS-CSF samples as described previously [5]. In brief, 2.5 μg of total protein was added to 150 μl of 22 μmol 4-methylumbelliferyl β-D-N,N′,N′-triacetylchitotrioside hydrate (Sigma-Aldrich, USA), prepared in 0.5 M citrate-phosphate buffer (pH 5.2). Following incubation for 15 min at 37 °C, the reaction was stopped using 100 μl of 0.5 mol Na_2_CO_3_-NaHCO_3_ buffer (pH 10.7). The fluorescence was captured at 365-nm excitation and 450-nm emission using Tecan 2500 fluorimeter (Tecan, USA), and the data was expressed as micromoles of substrate hydrolyzed/μl/min.

### CHIT-1 dosage for in vivo studies

The dosage for intrathecal injection of CHIT-1 was based upon the average amount of CHIT-1 present in 5 μl of CSF (approximately 90 pg of CHIT-1 as determined by ELISA). The following doses of CHIT-1 were used: 50 pg, 100 pg, 200 pg and 500 pg.

### In vivo studies

Neonatal Wistar rat pups were obtained from Central Animal Research Facility (CARF), NIMHANS, Bangalore, after obtaining clearance from Institutional Animal Ethics Committee (IAEC). Rat neonates along with lactating mothers were housed at an ambient temperature of 26 ± 2 °C and subjected to the routine light/dark cycle. Lactating mothers had ad libitum access to food and water. Post-natal day 3 pups were intrathecally injected with 5 μl of buffer, different doses of recombinant human CHIT-1, N-CSF and ALS-CSF [20–22]. Briefly, rat pups were anesthetized with halothane, and a dorsal midline incision (1 mm) was made about 1 cm rostral to the base of the tail. Samples were injected into the subarachnoid space with the aid of a micro-injector at a flow rate of 400 nl/min. The needle was retained in its place for 1–2 min following injection to prevent back flow of injected sample. The incision was cleaned, sutured and an anti-inflammatory agent, Healex, was sprayed on to the sutures. Pups were allowed to recover from anaesthesia and housed with the mother. Pups of both genders were randomly assigned to each of the experimental groups.

### Immunohistochemistry

After 48 h of intrathecal injection, animals were anaesthetized with halothane and perfused transcardially using 4% paraformaldehyde [22, 23]. Spinal cords were dissected out, post-fixed in the same fixative for 24 h and meninges removed. Lumbar region cryoprotected with 30% sucrose was sectioned at 40-μm thickness using a cryostat (Leica, Germany).

For immunostaining, antigen unmasking was performed by incubating in sodium citrate buffer (10 mM sodium citrate, 0.05% tween 20, pH 6.0) for 5–10 min at 95 °C and blocked using 3% bovine serum albumin (BSA, Sigma) for 3 h. Sections were rinsed in 0.1 M PBST and then incubated in the first primary antibody. The sections were again washed and incubated with appropriate fluorescent-conjugated secondary antibody. Blocking was done with 3% BSA for 1 h and rinsed and treated subsequently with second primary antibody followed by incubation in second secondary antibody. For choline acetyl transferase (ChAT) staining, spinal cord sections were pretreated with ice cold methanol for 10 min. Lists of antibodies used are given in Table 2.

**Table 2:** List of antibodies used for immunohistochemistry

Primary antibody	Secondary antibody
Anti-Iba-1 goat polyclonal (1:400, Abcam), 48 h, 4 °C	Anti-goat FITC conjugated (1:200, Sigma), overnight, 4 °C
Anti-GFAP mouse monoclonal (1:400, MP Biomedicals), overnight, 4 °C	Anti-mouse Cy3 conjugated (1:200, Sigma), overnight, 4 °C
Anti-ChAT goat polyclonal (1:200, Millipore), 48 h, 4 °C	Anti-goat FITC conjugated (1:200, Sigma), overnight, 4 °C
Anti-phosphorylated neurofilament mouse monoclonal (1:800, SMI), overnight, 4 °C	Anti-mouse Cy3 conjugated (1:200, Sigma), overnight, 4 °C

*Iba-1* ionized calcium binding adaptor molecule 1, microglial marker, *GFAP* glial fibrillary acidic protein, astrocyte marker, *ChAT* choline acetyltransferase, motor neuron marker, *FITC* fluorescein isothiocyanate, *Cy3* cyanine3

The fluorescence images of the immunolabelled specimens were analysed using a laser confocal scanning microscope (Leica TCS-SL, Germany) with excitation at 488 and 514 nm for FITC and Cy3 respectively. Emission band width of FITC was maintained at 490–540 nm and 550–620 nm for Cy3 to prevent overlap of emission frequencies.

Analysis was performed on 10 sections per spinal cord, and 3 animals per group were used for the study. Number of ChAT positive motor neurons and Iba1 positive microglia in the ventral horn was counted, and the individual means were derived. Area and intensity of GFAP and SMI-31 in white matter was measured and quantified on a scale of 0 (minimum)–255 (maximum), using an in-built software.

### Nissl staining

Serial sections of the lumbar region of spinal cords post fixed in paraformaldehyde (40 μm) were taken on a vibratome (Leica, Germany), and every fourth section was stained with cresyl violet. Briefly, slides were dipped in chloroform for 1 min; passed in succession through 100% ethanol, 90% ethanol, 80% ethanol and 70% ethanol for 2 min; and rehydrated in distilled water for 5 min and then in cresyl violet for 2 min. Slides were allowed to differentiate in distilled water for 5 min, passed in succession through 70% ethanol for 20 s (× 2), 90% ethanol for 20 s (× 2), 100% ethanol for 1 min (× 2) and xylene for 1 min. The stained slides were dried and mounted with DPX. Images were captured at × 10 with a Leica BX 51 microscope. The number of motor neurons was quantified by using the particle analysis feature of ImageJ. Briefly, the images were converted to 8-bit binary with Huang thresholding, following noise removal and using a size-based filter above 250 μm^2^ for the analysis [24]. Data was obtained by analysing 5 sections per spinal cord, and 3 animals per group were used for the study.

### ELISA for pro-inflammatory molecules in tissue lysates

Lumbar region of spinal cord was dissected out 48 h post intrathecal injection from 3 animals per group. Tissues were snap frozen and stored at −80 °C till use. Samples containing 2% tissue lysate with 1% protease inhibitor were sonicated thrice at 15 Hz for 10 s. The lysates were centrifuged for 10 min at 5000×*g*, and the supernatant was used for ELISA. Samples were protein normalised, and 450 μg/ml of each sample was used. ELISA for IL-10 and TNFα was performed using commercially available kits (RayBiotech, USA) according to manufacturer’s protocol.

### Statistical analysis

Data was expressed as mean ± SEM. Statistical analysis was performed using the GraphPad Prism 6 and SPSS software. The data was analysed for significance using one-way ANOVA followed by Tukey’s post hoc test. Receiver operator characteristic (ROC) analysis was performed to assess optimal cutoff value and sensitivity and specificity of ELISA. MedCalc diagnostic test evaluation calculator was used to calculate predictive values. Pearson correlation was used to find possible correlation between CHIT-1 activity and disease severity on one hand and CHIT-1 level on the other hand. Spearman’s correlation was performed to assess correlation between CHIT-1 level and disease duration. It was also performed to correlate enzyme activity of CHIT-1 on one hand and disease severity as well as duration on the other hand. Partial correlation was used to find the relationship between CHIT-I level and disease severity and disease duration.

## Results

### CHIT-1 level is increased in CSF of ALS patients

**Fig. 1:**
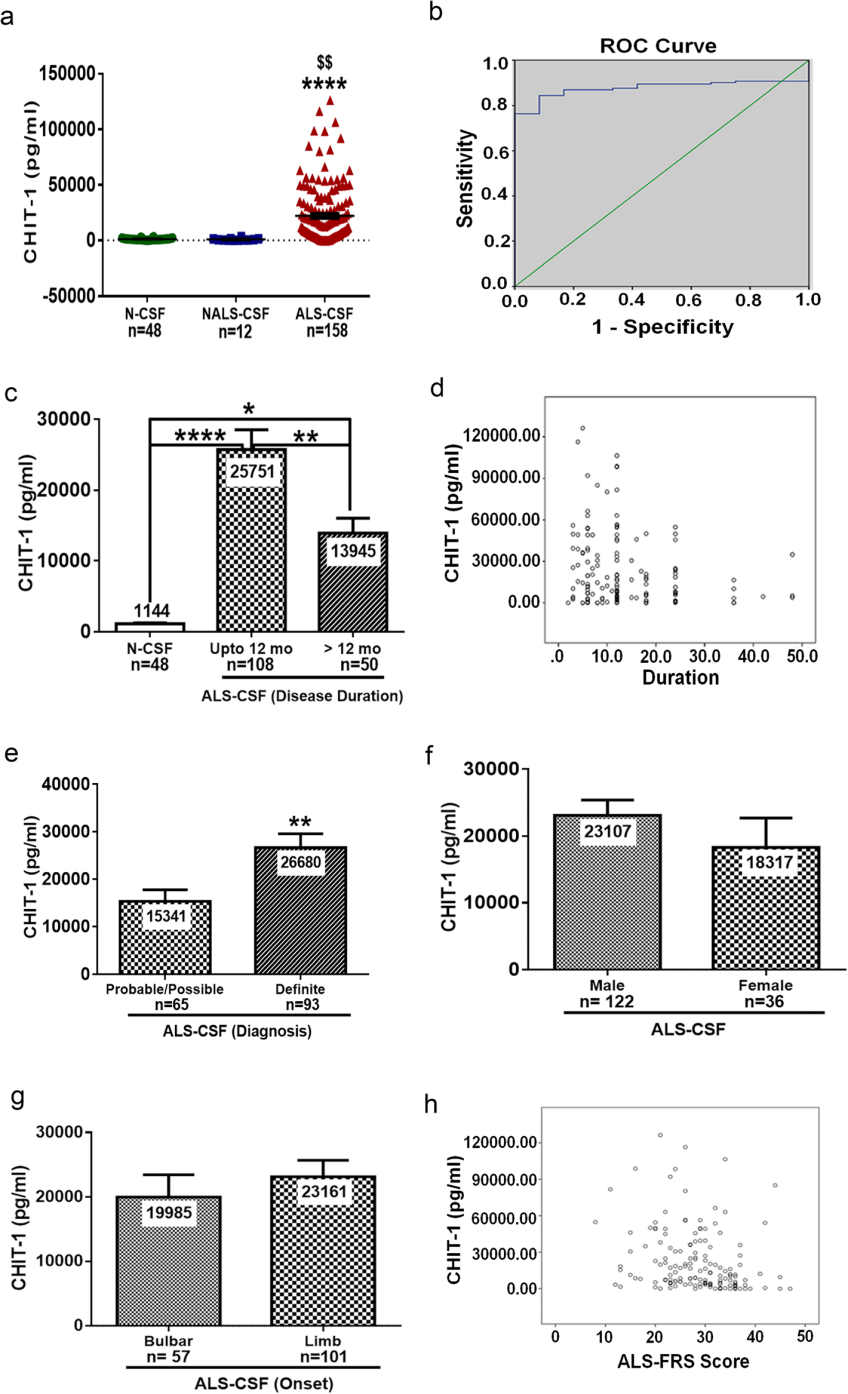
CHIT-1 levels in CSF of ALS patients and controls. ELISA showed increased levels of CHIT-1 compared to N-CSF and NALS-CSF (****p < 0.0001 vs. N-CSF and ^$$^*p* < 0.01 vs NALS-CSF) (**a**). Receiver operating curve (ROC) analysis (**b**). Increased levels of CHIT-1 in CSF of patients with lesser disease duration (****p < 0.0001 N-CSF vs. ALS-CSF up to 12 months, *p < 0.05 N-CSF vs. ALS-CSF > 12 months and **p < 0.01 ALS-CSF up to 12 months vs. ALS-CSF > 12 months (**c**). Correlation between CHIT-1 levels and disease duration (**d**). Increased level of CHIT-1 in definite cases compared to probable/possible cases of ALS (**p < 0.01 probable/possible vs. definite) (**e**). No significant difference was noticed between male vs. female (**f**) and bulbar vs. limb onset (**g**) of ALS. Correlation between CHIT-1 levels and ALS-FRS score (**h**)

CHIT-1 level was increased by 19-fold in ALS-CSF samples compared to N-CSF and 24-fold when compared to NALS-CSF (****p < 0.0001 vs. N-CSF and ^$$^*p* < 0.01 vs. NALS-CSF). There was no significant difference in the level of CHIT-1 between NALS-CSF and N-CSF. The mean CHIT-1 level in the N-CSF was 1144 ± 763.30 pg/ml (*n* = 48) and in NALS-CSF was 909.4 ± 934.43 pg/ml (*n* = 12) whereas in the ALS-CSF the mean was 22,015 ± 25,804 pg/ml (*n* = 158) (Fig. 1a). A cutoff value of 1405.43 pg/ml in CHIT-1 could discriminate sporadic ALS cases (as determined by neurological evaluation) from controls. This cutoff had a sensitivity of 87% and specificity of 83.3% with area under the ROC curve as 0.877 (95% confidence interval 0.826–0.929; *p* < 0.001) (Fig. 1b). The test had a positive predictive value of 98.59% and a negative predictive value of 32.26%. CSF from patients with lesser disease duration (< 12 months) had significantly higher CHIT-1 levels compared to CSF from patients with more than 12 months disease duration (**p < 0.01 > 12 months vs. up to 12 months) (Fig. 1c). Further, CHIT-1 levels and disease duration was negatively correlated (*r* = − 0.175; *p = 0.029) (Fig. 1d). CHIT-1 levels were also more in patients diagnosed with definite ALS (**p < 0.01 definite vs. probable/possible) (Fig. 1e). No significant difference in CHIT-1 levels between samples of male and female patients as well as between bulbar and limb onset cases was observed (Fig. 1f, g). A negative correlation was observed between CHIT-1 level and ALS-FRS score (*r* = − 0.254; ***p = 0.001) indicating that patients with high disease severity have high level of CHIT-1 (Fig. 1h). The correlation between CHIT-1 level and ALS-FRS score was independent of disease duration.

### Enzyme activity of CHIT-1 is increased in CSF of ALS patients

**Fig. 2:**
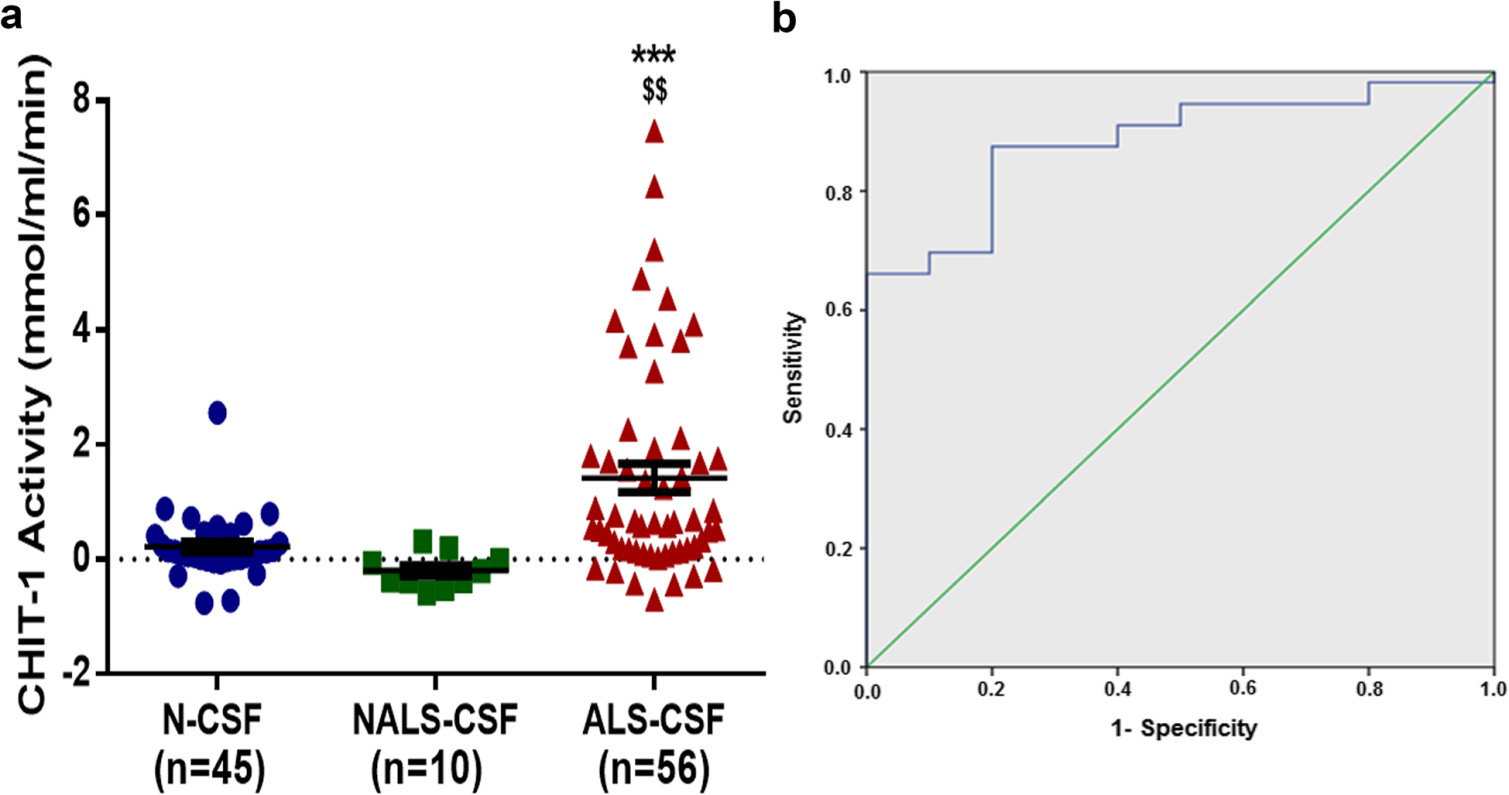
CHIT-1 enzyme activity in ALS-CSF and control CSF. Increased CHIT-1 activity in ALS-CSF compared to N-CSF and NALS-CSF (***p < 0.001 vs. N-CSF and ^$$^*p* < 0.01 vs. NALS-CSF) (**a**). Receiver operating curve (ROC) analysis (**b**)

The mean CHIT-1 activity in the N-CSF was 0.08537 ± 0.01760 μmol/μl/min (*n* = 45) and in NALS-CSF was 0.05149 ± 0.03538 μmol/μl/min (*n* = 10) whereas in the ALS-CSF the mean was 1.33 ± 0.2334 μmol/μl/min (*n* = 56). Thus, ALS-CSF samples had approximately 15-fold higher enzymatic activity when compared to N-CSF and 25-fold more when compared to NALS-CSF (***p < 0.001 vs N-CSF and ^$$^*p* < 0.01 vs NALS-CSF) The CHIT-1 activity did not differ significantly between NALS-CSF and N-CSF (Fig. 2a). A cutoff value of 0.1077989 μmol/μl/min can distinguish sporadic ALS cases with a sensitivity of 80.4% and specificity of 80%. (Area under the ROC curve = 0.882; 95% confidence interval 0.792–0.972; *p* < 0.001) (Fig. 2b). The test had a positive predictive value of 95.74% and a negative predictive value of 42.11%. No significant correlation was observed between CHIT-1 enzyme activity and disease duration (*r* = − 0.176; *p* = 0.196) or enzyme activity ALS-FRS score (*r* = 0.053; *p* = 0.696). A positive correlation was observed between CHIT-1 level and enzyme activity (*r* = 0.379; **p = 0.004). CHIT-1 levels and enzyme activity when used together can diagnose ALS patient with sensitivity of 100%, specificity of 88.89%, positive predictive value of 97.78% and negative predictive value of 100%.

### CHIT-1 activates glial cells in vivo

**Fig. 3:**
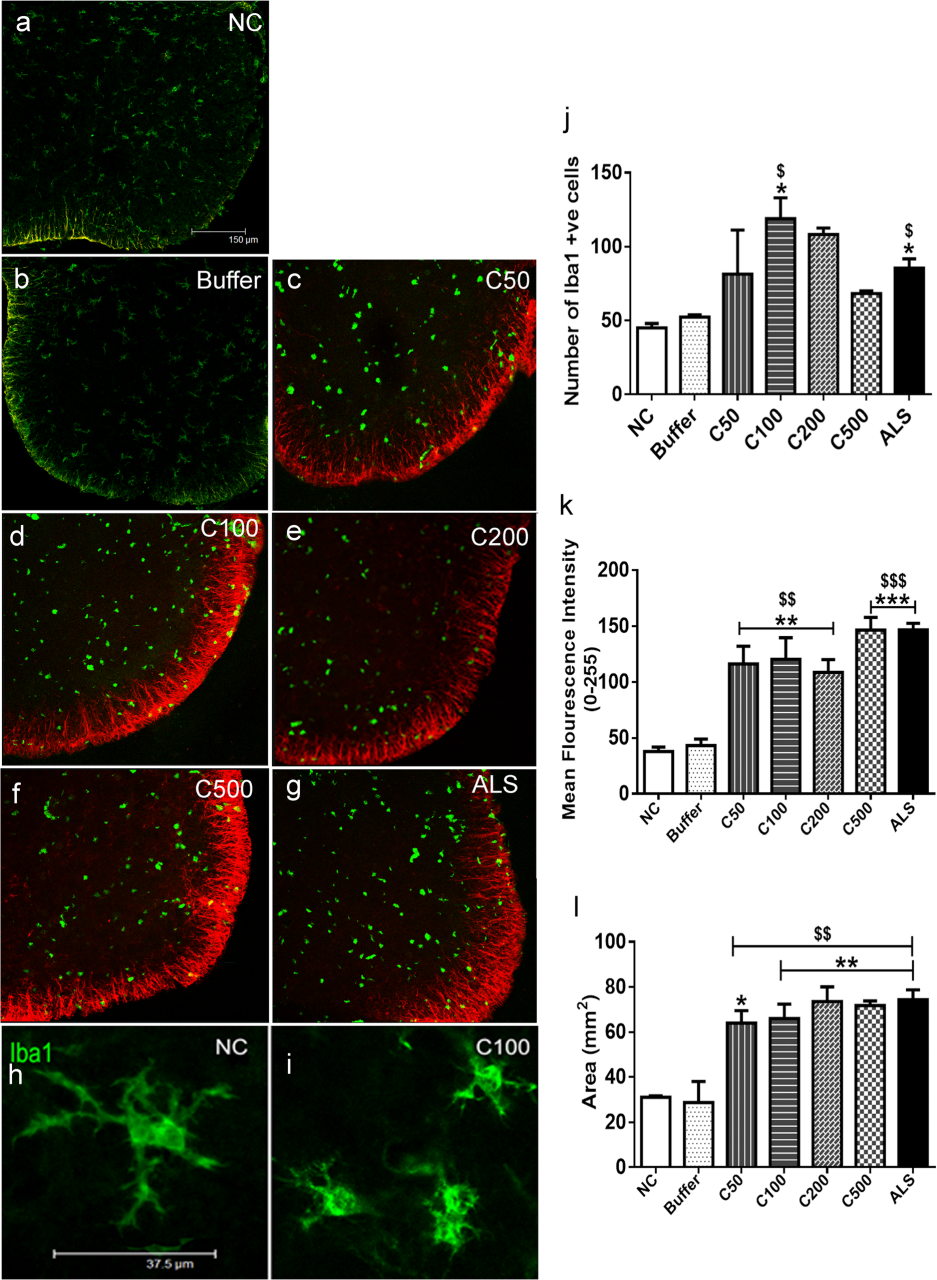
Effect of in vivo administration of CHIT-1 in glia. Representative confocal micrographs (merged) of spinal cord sections co-labeled with Iba1 (green) and GFAP (red) in normal control (NC; **a**), Buffer (**b**), CHIT-1 (**c**–**f**) and positive control (ALS, **g**) groups. Note the increase in Iba1 labelled microglia in CHIT-1 (mainly in C100) and ALS groups. An increase in GFAP expression was also observed in CHIT-1 (**c**–**f**) and ALS (**g**) groups. Note the change in morphology of the Iba1 labelled microglia from long process bearing ones in control (**h**) to those bearing short processes in CHIT-1 group (**i**). Scale bar = 150 μm (for all images). CHIT-1 induces a significant upregulation of Iba1 positive microglial cells at a dose of 100 pg similar to ALS-CSF group unlike buffer and normal controls (*p < 0.05 C100 and ALS vs. NC; ^$^*p* < 0.05 C100 and ALS vs. buffer) (**j**). Histogram representing enhanced expression of GFAP in ventral horn white matter in CHIT-1 group compared to buffer and normal controls in terms of intensity (**p < 0.01 C50, C100, C200 vs. NC; ***p < 0.001 C500, ALS vs. NC and ^$$^*p* < 0.01 C50, C100, C200 vs. buffer; ^$$$^*p* < 0.001 C500 and ALS vs. buffer) (**k**) and area (*p < 0.05 C50 vs. NC; **p < 0.01 C100, C200, C500, ALS vs. NC and ^$$^*p* < 0.01 NC vs. C50, C100, C200, C500 and ALS) (**l**)

**Fig. 4:**
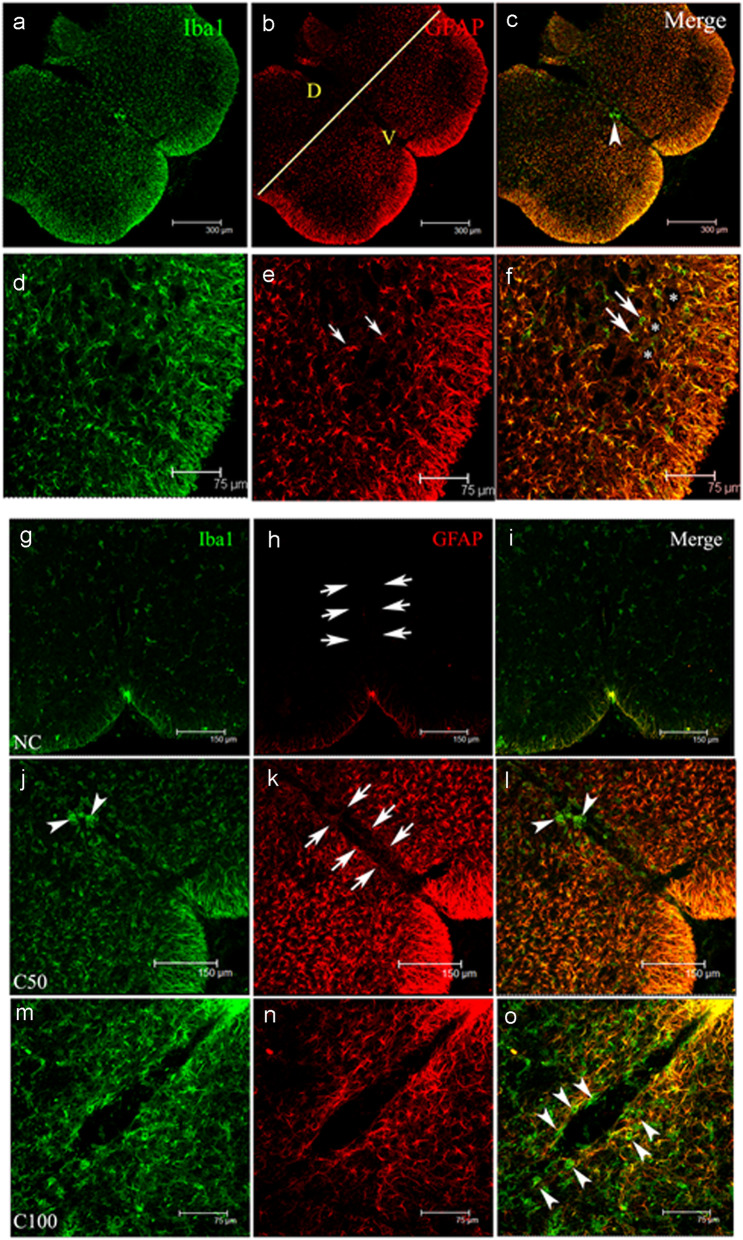
Effect of CHIT-1 on glia in different regions of the spinal cord. Representative confocal micrographs showing Iba1 (green) and GFAP (red) expression in the spinal cord sections (**a**–**f**). Significant amount of gliosis was noted in the ventral horn (V) compared to dorsal horn (D). The yellow coloured line demarcates the areas (**b**). The higher magnification images show the presence of astrocytes (red, small arrows, (**e**)) in the grey matter and also the Iba1 (green, big arrows, (**f**)) immunoreactive microglia surrounding the motor neurons (*). Scale bars are indicated. Representative confocal photomicrographs showing Iba1 (green) and GFAP (red) expression around the central canal (arrows) (**g**–**o**). Note the faint staining in normal control (NC, (**g**, **h**, **i**)) whereas both the markers show upregulation in the 50 and 100 pg concentrations of CHIT-1 (C50 and C100) (**j**–**o**). Note the presence of amoeboid shaped microglia within the vicinity of the canal (arrowheads) (**o**). Scale bars are indicated

Intrathecal administration of CHIT-1 for 48 h resulted in an increase in Iba1 positive microglial cells in the ventral horn of spinal cords, where a peak was observed at 100 pg of CHIT-1. ALS-CSF also elicited a similar response (*p < 0.05 C100 and ALS vs. NC; ^$^*p* < 0.05 C100 and ALS vs. buffer) (Fig. 3a–i, j). In addition, intrathecal administration of human recombinant CHIT-1 induced a significant activation of astrocytes. Increase in the mean fluorescent intensity of GFAP immunoreactivity was noted in the white matter of the ventral horn of the spinal cord of pups injected with 50, 100, 200 and 500 pg CHIT-1 in comparison with pups injected with buffer (vehicle control) and normal control (NC). GFAP immunoreactivity was higher in ALS-CSF-injected spinal cord sections compared to NC and buffer (**p < 0.01 C50, C100, C200 vs. NC; ***p < 0.001 C500, ALS vs. NC and ^$$^*p* < 0.01 C50, C100, C200 vs. buffer; ^$$$^*p* < 0.001 C500 and ALS vs. buffer) (Fig. 3a–g, k). The area of GFAP staining in the white matter also increased significantly in the spinal cords after administration of CHIT-1 or ALS-CSF (*p < 0.05 C50 vs. NC; **p < 0.01 C100, C200, C500, ALS vs. NC and ^$$^*p* < 0.01 buffer vs. C50, C100, C200, C500 and ALS) (Fig. 3a–g, l). Taken together, the observed results show that exposure of the spinal cord to CHIT-1 causes microgliosis and astrogliosis in vivo, specifically in the ventral horn while the dorsal horn being spared (Fig. 4a–f).

### Effect of CHIT-1 on central canal pathology

Qualitative observations revealed the presence of several amoeboid-shaped Iba1 immunopositive cells around the central canal. A number of GFAP immunopositive astroglia bearing long processes were also seen to infiltrate the central canal following CHIT-1 administration (Fig. 4g–o). These changes were observed irrespective of the dose strength of CHIT-1 albeit as low as 50 pg/ml. Further investigations need to be carried out to determine the precise cause for such intense gliosis around the central canal.

### CHIT-1 enhances proinflammatory molecules in vivo

**Fig. 5:**
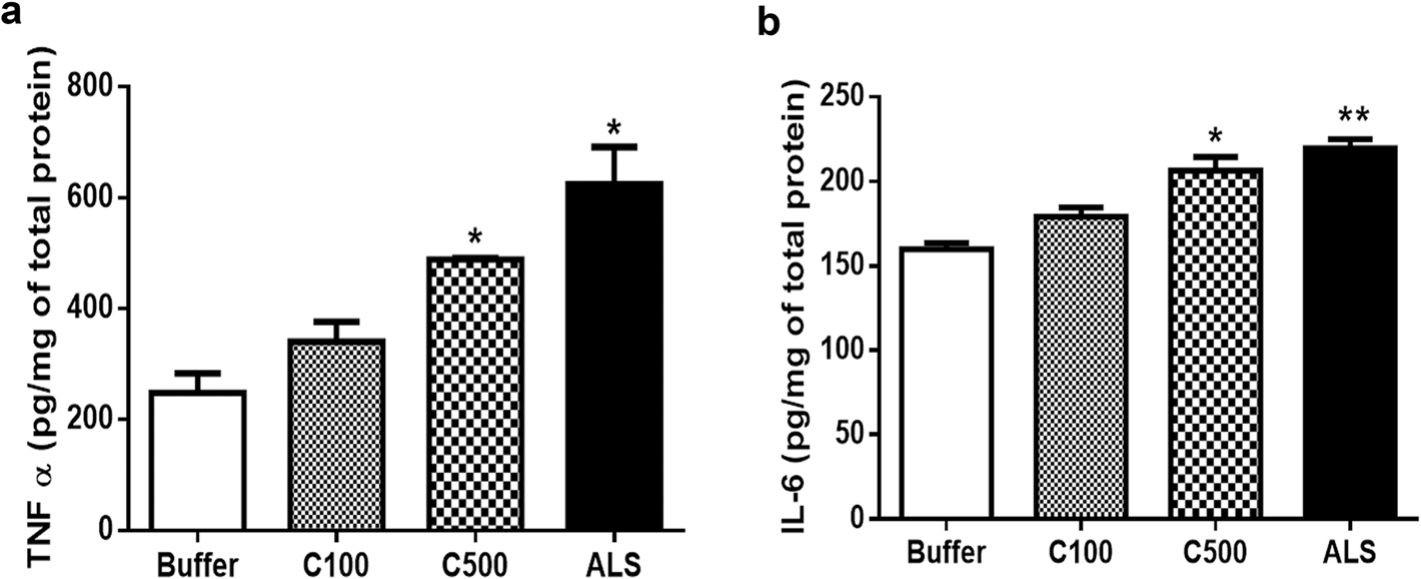
Effect of CHIT-1 on proinflammatory molecules. Histogram representing enhanced level of TNFα in lumbar spinal cord lysates of CHIT-1 (500 pg) and ALS group and compared to buffer control (*p < 0.05 C500, ALS vs. buffer) (**a**). Increased level of IL6 in lumbar spinal cord lysates of CHIT-1 (500 pg) and ALS group and compared to buffer control (*p < 0.05 C500 vs. buffer; **p < 0.01 ALS vs. buffer) (**b**)

The levels of TNFα, a proinflammatory molecule, was increased in tissue lysates from the lumbar region of the spinal cord of pups injected with CHIT-1 at a higher dose, i.e. 500 pg and ALS-CSF (*p < 0.05 C500 and ALS-CSF vs Buffer) (Fig. 5a). Similar changes were also observed in the levels of IL-6 (*p < 0.05 C500 vs buffer; **p < 0.01 ALS vs. buffer (Fig. 5b).

### CHIT-1 induces neuronal loss in vivo

**Fig. 6:**
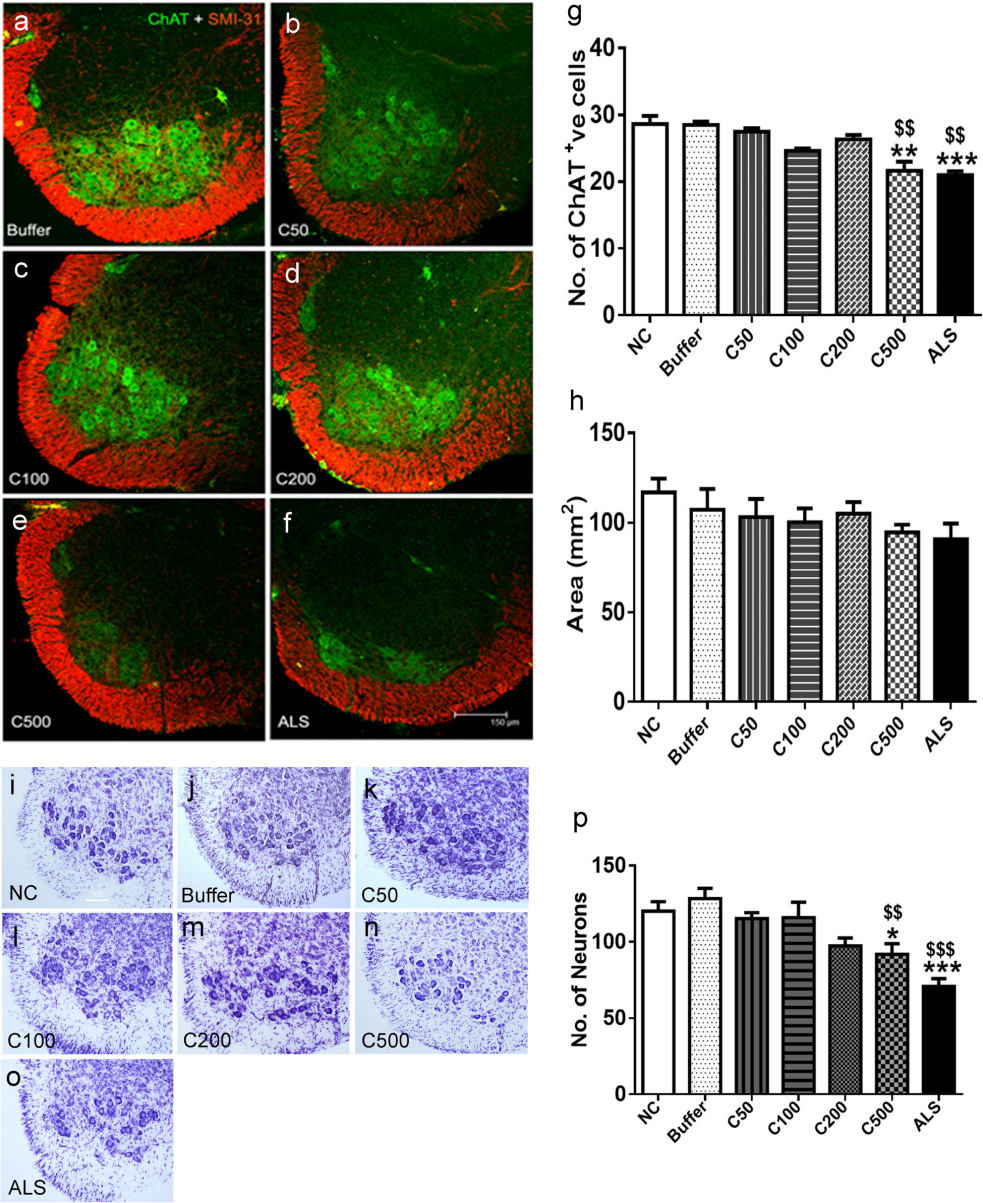
Effect of CHIT-1 on motor neurons and axonal density. Representative confocal images (merged) of spinal cord sections co-labeled for ChAT (green) and SMI-31 (red) in control [buffer, (**a**)], CHIT-1 [C50, 100, 200, 500 pg; (**b**–**e**)] and also ALS-CSF injected (**f**) groups. Scale bar = 150 μm (for all images). CHIT-1 induces loss of ChAT positive motor neuronal cells, similar to ALS-CSF, at a dose of 500 pg. (**p < 0.01 C500 vs. NC; ***p < 0.001 ALS vs. NC; ^$$^*p* < 0.01 C500 and ALS vs. buffer) (**g**). Histogram representing area of phosphorylated neurofilaments (SMI-31) in white matter of ventral horn of spinal cord (**h**). Representative images of Nissl-stained spinal cord sections of normal control [NC, (**i**)], Buffer (**j**), CHIT-1 [C50, C100, C200, C500 pg; (**k**–**n**)] and ALS-CSF (**o**) injected groups. CHIT-1 (500 pg) and ALS-CSF results in motor neuronal loss (*p < 0.05 C500 vs NC; ***p < 0.001 ALS vs. NC; ^$$^*p* < 0.01 C500 vs buffer; ^$$$^*p* < 0.001 ALS vs. buffer) (**p**)

A reduction in the number of ChAT immunopositive neurons in the spinal cord was observed upon intrathecal administration of CHIT-1 at a higher dose, i.e. 500 pg. This was similar to the cell loss observed in response to ALS-CSF (**p < 0.01 C500 vs NC; ***p < 0.001 ALS vs. NC; ^$$^*p* < 0.01 C500, ALS vs. buffer) (Fig. 6a–f, g). Nissl staining performed to confirm the loss of motor neurons corroborated the ChAT immunostaining data. Significant reduction in the number of motor neurons was observed in pups injected with C500 pg and ALS-CSF compared to controls (*p < 0.05 C500 vs NC; ***p < 0.001 ALS vs. NC; ^$$^*p* < 0.01 C500 vs buffer; ^$$$^*p* < 0.001 ALS vs. buffer) (Fig. 6i–o, p). There was a trend in reduction in the white matter immunoreactivity of phosphorylated neurofilaments following administration of 500 pg of CHIT-1 (Fig. 6a–f, h).

## Discussion

We have previously reported that CHIT-1 level was higher in ALS-CSF by using proteomics and ELISA in a small sample size of 16 ALS patients [5]. Following our report, increased CHIT-1 level in ALS patients has been confirmed by other studies as well [6, 14, 18]. In the current study, we have demonstrated the increased levels of CHIT-1 in a large cohort of 158 samples. We observed a significant negative correlation between CHIT-1 levels and disease duration, i.e. higher levels of CHIT-1 in CSF of patients with shorter history of the disease. Similar trend was reported by Thompson et al. where a moderate correlation of CHIT-1 level to disease progression was observed [18]. Hence, we can conclude that CHIT-1 can be used for early and accurate diagnosis of ALS, as our study could discriminate ALS patients from controls with a cutoff value of CHIT-1 as low as 1405.43 pg/ml with a sensitivity of 87% and specificity of 83.3%. Steinacker et al. also reported a similar cutoff value of 2003 pg/mL with sensitivity of 87% and specificity of 84% [6]. Chen et al. reported a cutoff of 1593.779 ng/L with a sensitivity of 83.8% and specificity of 81.1% [14]. The similar cutoff values in these studies across the ethnic groups validate the use of CHIT-1 as a biomarker of ALS amongst various populations. The positive correlation between CHIT-1 level and disease severity further strengthens its use in diagnosis.

We were also the first to report increased CHIT-1 enzyme activity in ALS-CSF. The 15-fold increase in enzyme activity of CHIT-1 in the current study with larger cohort further proves its use in diagnosis of ALS as it has a sensitivity of 80.4% and specificity of 80%. The increase in activity can be due to high level of protein present as a positive correlation was observed between enzyme activity and the levels of CHIT-1. This is the only study which has integrated both CHIT-1 level and its enzyme activity. The positive and negative predictive values (97.78% and 100% respectively) observed when both CHIT-1 level and enzyme activity were considered together suggest that combining the two parameters can significantly improve diagnostic accuracy. When both ELISA and enzyme activity of CHIT-1 is considered, the sensitivity is 100% and specificity is 88.89%, which can reduce the false positive and false negative results.

However, the consequence of CHIT-1’s activity, its presence in the CSF and its exact role in ALS pathogenesis remain to be determined. Accumulation of glucose and glucosamine via impaired glucose metabolism along with activation of hexosamine pathway can lead to formation of chitin-like polymers in the brain which have been detected in AD patients [25, 26]. Interestingly, ALS patients exhibit impaired glucose tolerance [27]. It can be hypothesised that the excess glucose in circulation taken up by the brain could accumulate in the neurons leading to the formation of glucosamine and its polymers which can serve as alternate substrates for CHIT-1.

The increased levels of CHIT-1 in ALS-CSF and its enhanced expression by microglial cells upon exposure to ALS-CSF persuaded us to investigate its non-chitin role in ALS pathogenesis. CHIT-1 was able to activate glial cells in the spinal cord of Wistar rat pups. The presence of reactive microglial cells is reported in the cortex, brain stem and spinal cord of the post-mortem samples of ALS patients [28]. PET imaging of ALS patients also reported increased microglial proliferation corresponding to its activation in the cortex [29, 30]. Addition of ALS-CSF to pure microglial cultures also resulted in microglial proliferation and activation. The activated microglia were primed to attain a toxic phenotype with increased release of reactive oxygen species and pro-inflammatory molecules [31]. In our observation, the proliferation of microglia was notable following exposure to CHIT-1 at a concentration found in ALS-CSF samples.

Increase in amoeboid-shaped microglial cells observed in the ventral horn of spinal cord in CHIT-1 injected group suggests microglial differentiation and they acquire a phenotype with enlarged soma and retracted processes. The presence of microglial cells in the vicinity of the central canal is intriguing. While we are unable to determine the precise cause for this occurrence, it is possible that it might be the result of blood brain/blood spinal cord barrier (BBB/BSCB) breach induced by CHIT-1. CHIT-1 supplementation in an in vitro model of BBB resulted in an increased migration of peripheral blood mononuclear cells across human brain micro-vascular endothelial cells [15]. Our preliminary findings of enhanced serum S100β levels in CHIT-1 injected pups controls (*p < 0.05 CHIT-1 vs normal control; data not shown, unpublished observations) also hint towards its possible role in BBB breach as increased serum S100β levels act as marker for the same [32, 33]. However, further experiments are required to prove whether CHIT-1 is able to induce BBB/BSCB breach in our model.

The significant increase in the immunoreactivity for GFAP in the ventral spinal cord in response to CHIT-1 may suggest proliferation and activation of astrocytes. Additionally, the GFAP reactivity was particularly intense in the CHIT-1-exposed group compared to controls which were almost GFAP negative. A similar feature was also observed while comparing the dorsal and ventral horns, wherein the latter area was significantly more gliotic, which suggests selective vulnerability of ventral horn (motor neurons) compared to dorsal horn (sensory neurons). Gliosis appeared prominent, even with the lower doses of CHIT-1, and the effect persisted at higher doses as well. Both CHIT-1 and ALS-CSF appeared to produce similar astroglial responses. This result corroborates with our earlier findings of ALS-CSF-induced enhancement of GFAP expression in both grey and white matter of neonatal rat spinal cord [34] and elevated levels of S100β and decreased GLT-1 expression in astrocytes grown in spinal cord cultures. Transformation in the morphology of astrocytes from flat to process bearing was also observed upon exposure to ALS-CSF [21]. The aberrant astrocytes possessing high proliferation capacity are also observed in the spinal cord of human autopsy specimens and that of SOD1 mice where they are shown to secrete soluble factors which induce neuronal death [35]. Ependymal cells lining the central canal harbour cells with stem cell properties. These cells give rise to astrocytes in response to stroke or spinal cord injury [36, 37]. This phenomenon could explain the presence of astrocytes around central canal in animals injected with CHIT-1 or ALS-CSF and the extension of astrocytic process to central canal in ALS-CSF group.

The intricate cellular interplay between microglia and astrocytes could be a major factor leading to neurodegeneration. Several studies have shown non-cell autonomous degeneration of motor neurons [1, 4]. The disease progression was delayed in the mice in which mutant SOD1 was specifically deleted from microglia or astrocytes [38]. When wild type neurons were co-cultured with astrocytes or microglia expressing mutant SOD-1 protein, neuronal loss was observed, suggesting that glial activation is pre-requisite in ALS disease pathogenesis [39]. It is well-known that microglial activation is a primary event in response to any injury or damage to CNS. Microglial proliferation was observed from the pre-clinical stage of disease in mSOD1 rats while hypertrophic GFAP-labelled astrocytes were seen only during the late clinical stage [40]. Microglial cells responded prior to astrocytes when exposed to ALS-CSF as microglia showed increased release of pro-inflammatory molecules like interleukin 6, TNF-α and glutamate as early as 12 h while astrocytes showed a similar response only after 24 or 48 h [31, 41].

**Fig. 7:**
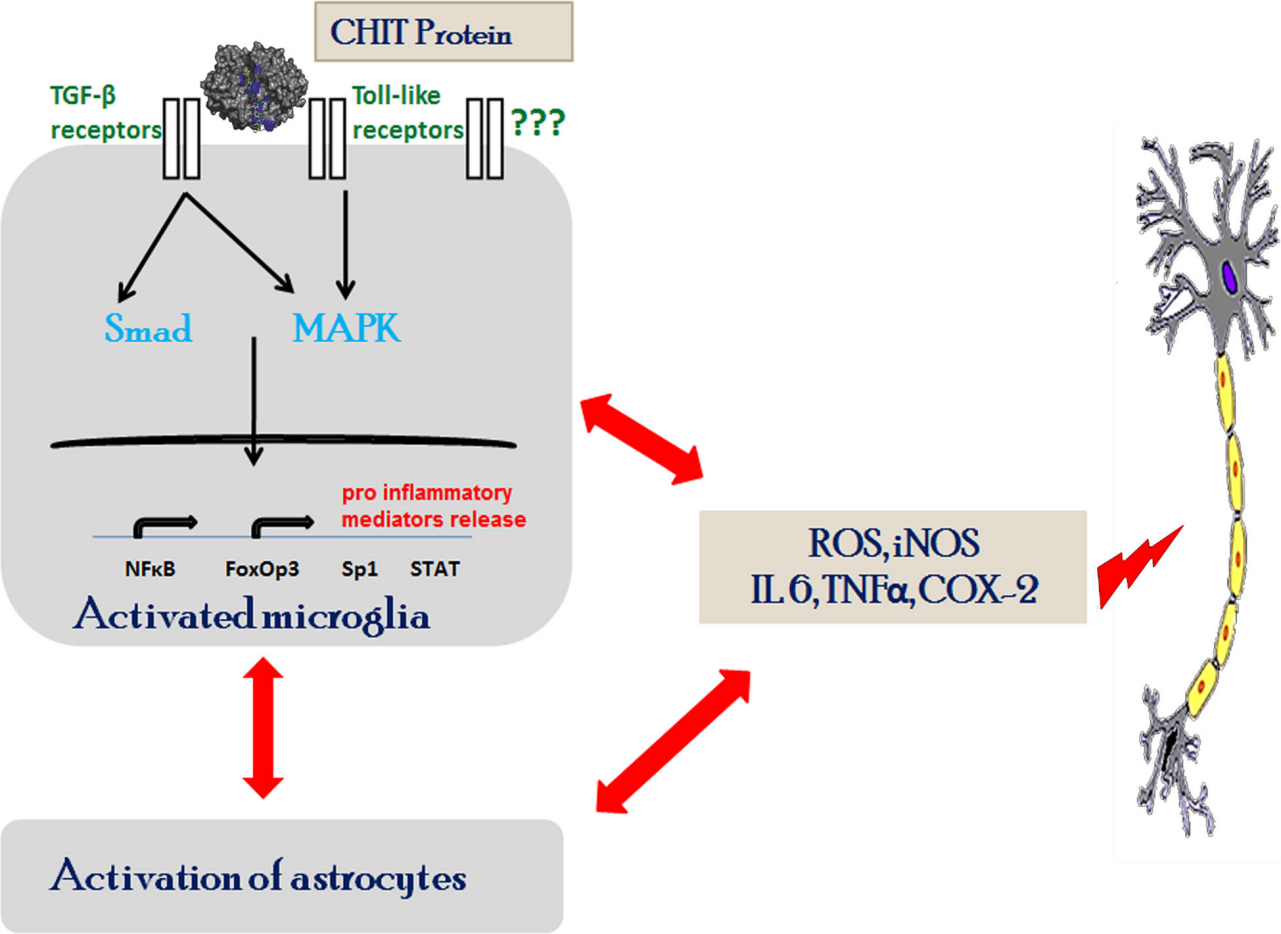
Hypothesis of mechanisms of CHIT-1 induced neurotoxicity in ALS. CHIT-1 activates microglial cells by possibly interacting with receptors (e.g. transforming growth factor beta (TGF-β) or toll-like receptors) leading to the release of proinflammatory mediators. This leads to the heightened glial response with an increase in reactive astrocytes and activated microglia. Neurotoxic cytokines like IL-6, TNF-α along with ROS and nitric oxide released by both these glial cell types result in motor neuronal degeneration

CHIT-1 was able to induce microglial proliferation and release of microvesicular structures while it did not exert any significant effect on primary astrocytic cultures [31]. The induction of microgliosis and astrogliosis in the ventral horn by CHIT-1 signifies the active participation of CHIT-1 in the initiation and progression of neuroinflammatory process. CHIT-1 administration resulted in increased levels of proinflammatory molecules in spinal cord lysates. TNFα and IL-6 was reported to be elevated in CSF and serum of ALS patients [42, 43]. The proinflammatory molecules sustain the inflammatory process whose effect can be mitigated by inhibiting their signalling pathways [44]. Investigating glial transformation to toxic phenotype, temporal profiling of glial activation and the pathways altered upon CHIT-1 administration would give better insights on the mechanisms behind CHIT-1-induced neuroinflammation. A decrease in motor neurons was observed in the spinal cord of pups injected with high dose of CHIT-1. This can be due to the increased release of proinflammatory molecules by activated glia. CHIT-1-induced heightened neuroinflammatory response could be the reason for high severity in patients with high CHIT-1 levels. Olsson et al. have shown that CHIT-1 is extremely stable in CSF [45]. Therefore, it might also be possible that prolonged presence of CHIT-1 might cause neurodegeneration. We have earlier reported that ALS-CSF induced apoptosis and reduced ChAT expression in NSC-34 cells [46] with altered neuronal activity and motor deficits in adult rats [47, 48]. Conditioned medium from glial cells exposed to ALS-CSF also resulted in reduced viability of NSC-34 cells [31, 41]. The reduction in the white matter area observed with staining by phospohorylated neurofilaments can be correlated to white matter atrophy observed in ALS patient spinal cord [49]. Based on these data, it could be assumed that CHIT-1 primarily activates microglia while astrocytes respond to activated microglia and join to evoke a sustained toxicity resulting in the release of proinflammatory molecules, which can further lead to neuronal death (Fig. 7).

## Conclusion

We hypothesize that CHIT-1-induced microgliosis and astrogliosis might be one of the critical events that lead to the motor neuron degeneration. Our study demonstrates CHIT-1 not only as an early diagnostic biomarker for ALS, but also as a key molecule for therapeutic intervention targeting neuroinflammation at the early stages of this crippling and fatal neurodegenerative disorder.

## Data Availability

The datasets generated during and/or analysed during the current study are available from the corresponding author on reasonable request.

## Abbreviations

ALS-CSF: Cerebrospinal fluid from amyotrophic lateral sclerosis patients
ALS-FRS: ALS Functional Rating Scale
BBB/BSCB: Blood brain/blood spinal cord barrier
ChAT: Choline acetyltransferase
CHIT-1: Chitotriosidase
Cy3: Cyanine3
FITC: Fluorescein isothiocyanate
GFAP: Glial fibrillary acidic protein
Iba-1: Ionized calcium binding adaptor molecule 1
NALS-CSF: CSF from age and gender matched patients with non-neurodegenerative, non-infectious neurological diseases
N-CSF: Normal CSF (CSF of patients undergoing orthopaedic surgery without any neurological involvement)
TNF-α: Tumour necrosis factor alpha
